# Estimated Infection and Vaccine Induced SARS-CoV-2 Seroprevalence in Israel among Adults, January 2020–July 2021

**DOI:** 10.3390/vaccines10101663

**Published:** 2022-10-05

**Authors:** Ravit Bassal, Lital Keinan-Boker, Dani Cohen, Ella Mendelson, Yaniv Lustig, Victoria Indenbaum

**Affiliations:** 1 Israel Center for Disease Control, Ministry of Health, Gertner Institute, Chaim Sheba Medical Center, Tel-Hashomer 52621, Israel; 2 Department of Epidemiology and Preventive Medicine, Sackler Faculty of Medicine, Tel-Aviv University, Tel-Aviv 69978, Israel; 3 School of Public Health, University of Haifa, Haifa 3498838, Israel; 4 Central Virology Laboratory, Public Health Services, Ministry of Health, Chaim Sheba Medical Center, Tel Hashomer 52621, Israel

**Keywords:** SARS-CoV-2, antibodies, Israel, National Sera Bank, Receptor binding domain, seroprevalence

## Abstract

Severe Acute Respiratory Syndrome Coronavirus 2 (SARS-CoV-2) emerged in Israel in February 2020 and spread from then. In December 2020, the FDA approved an emergency use authorization of the Pfizer-BioNTech vaccine, and on 20 December, an immunization campaign began among adults in Israel. We characterized seropositivity for IgG anti-spike antibodies against SARS-CoV-2 between January 2020 and July 2021, before and after the introduction of the vaccine in Israel among adults. We tested 9520 serum samples, collected between January 2020 and July 2021. Between January and August 2020, seropositivity rates were lower than 5.0%; this rate increased from September 2020 (6.3%) to April 2021 (84.9%) and reached 79.1% in July 2021. Between January and December 2020, low socio-economic rank was an independent, significant correlate for seropositivity. Between January and July 2021, the 40.00–64.99-year-old age group, Jews and others, and residents of the Northern district were significantly more likely to be seropositive. Our findings indicate a slow, non-significant increase in the seropositivity rate to SARS-CoV-2 between January and December 2020. Following the introduction of the Pfizer-BioNTech vaccine in Israel, a significant increase in seropositivity was observed from January until April 2021, with stable rates thereafter, up to July 2021.

## 1. Introduction

Severe Acute Respiratory Syndrome Coronavirus 2 (SARS-CoV-2) was first diagnosed in December 2019 in Wuhan, China, and from January 2020 the virus spread to other provinces in China [1]. From February 2020, SARS-CoV-2 spread worldwide, and on 21 February, Israel confirmed the first case of COVID-19. Since then, the virus has spread throughout the country [1].

Non-pharmaceutical preventive measures were applied in Israel to control the spread of the virus, including social distancing, education institute closures, face masks, and limitations on traveling in and out of the country. These measures demonstrated high effectiveness in reducing morbidity [2], though a long-term solution was required.

On December 11, 2020, Pfizer received an Emergency Use Authorization (EUA) for the BNT162b2 mRNA vaccine (Pfizer-BioNTech), and the immunization campaign began in Israel on 20 December. Of 6,480,700 citizens aged 16 years and older in Israel [3], by 31 July 2021, 5,616,678 (86.7%) were administered with the first dose, 5,253,773 (81.1%) with the second dose, and 15,272 (0.2%) with the third dose (booster) [4].

Since December 2020, several variants have emerged and have been classified as variants of concern (VOCs) by the World Health Organization (WHO): Alpha (B.1.1.7, first observed in the United Kingdom, Date of Designation (DoD): December 2020); Beta (B.1.351, first observed in South Africa, DoD: December 2020); Gamma (P.1, first observed in Brazil, DoD: January 2021), and Delta (B.1.617.2, first observed in India, DoD: May 2021) [5]. These variants contain various mutations on the spike protein, are highly transmissible, and demonstrated a degree of escape from protective antibody induced by natural infection or, to a lesser degree, after immunization [6].

Sero-surveys conducted around the world and in Israel mostly examined the seroprevalence against SARS-CoV-2 only over a short period. Information on the dynamics of SARS-CoV-2 seropositivity is required to provide insights on the population exposure to the virus in the pre-vaccination era, and the effect of the immunization on the population.

The objectives of the study were to estimate the seropositivity rate to anti-SARS-CoV-2 antibodies due to infection between January and December 2020 and due to infection and vaccination between January and July 2021 in Israel, and to identify associated risk factors for seropositivity in these periods.

## 2. Materials and Methods

Sampling: Residual sera samples of adults aged 16 years and older were obtained from the Israel National Sera Bank (INSB) established in 1997 in the Israel Center for Disease Control. The samples were collected from individuals who performed routine or diagnostic blood tests between January 2020 and July 2021 in the following laboratories: the Soroka laboratory of the Clalit Health Maintenance Organization (HMO) (non-hospitalized patients) in the south of Israel, the Haifa and Western Galilee laboratory of the Clalit HMO in northern Israel, the Jerusalem Clalit HMO in Central Israel, the Mayanei Hayeshua Medical Center in Bnei-Brak, and the National Blood Service (NBS) of the Medical Emergency Services in Israel (Magen David Adom) in central Israel. The samples were collected throughout the period, aliquoted and stored at a temperature of −80 °C. For each sample, the retrieved data were age, gender, district of residence (North, Haifa, Central, Tel Aviv, Jerusalem, South, and Judea and Samaria), birth country (Israel vs. other), population group (Jews and others, and Arabs), and socio-economic rank. The data on socio-economic rank were based on city of residence, and an index published by the Central Bureau of Statistics that ranks the authorities in Israel based on a wide range of variables and ranges between 1 (the lowest) and 10 (the highest) [7]. Other than these details, the samples are anonymized. Further details regarding the sera collection and the INSB representativeness were described previously [8].

Ethics: Sera sample collection is approved by the legal department of the Israeli Ministry of Health.

Laboratory methods: The samples were tested for SARS-CoV-2-specific IgG antibodies using an in-house Enzyme-Linked Immunosorbent Assay (ELISA) based on the Receptor-Binding Domain (RBD) of the Spike protein [9]. Using a sample cut-off of 1.1, a lower value was defined as negative, and equal or higher value was defined as positive. The test sensitivity and specificity were 88% and 98%, respectively [9]. The samples were tested in the Central Virology Laboratory of the Ministry of Health, located in Sheba Medical Center.

Data analysis: Seropositivity rates were calculated by dividing the number of positive samples to SARS-CoV-2 IgG antibodies by the number of tested samples, and 95% confidence intervals (CI) were calculated. Across the study period, we defined two periods according to the vaccine introduction in Israel. The first period was between January and December 2020, before the vaccine was available, and the second period was between January and July 2021, after the introduction of the vaccine. For each period, seropositivity rates were calculated according to demographic characteristics, and univariable and multivariable analyses were applied, to further evaluate the association between demographic characteristics and seropositivity. Unadjusted and adjusted odds ratio (OR) and 95% confidence intervals (CI) were calculated, accordingly.

Statistical significance was determined at a level of *p*-value < 0.05. The statistical analyses were performed using the SAS Enterprise Guide software (version 7.12, SAS Institute Inc., Cary, NC, USA). Trends in seropositivity rates were expressed using the monthly percent change (MPC) calculated by Joinpoint software (Joinpoint Regression Program, Version 4.9.0.0, March 2021; Statistical Research and Applications Branch, National Cancer Institute. Calverton, MD, USA).

## 3. Results

Between January 2020 and July 2021, 9520 sera bank samples of Israeli 16+ year olds were collected. Table 1 shows the demographic characteristics of the total study population by period.

**Figure 1:**
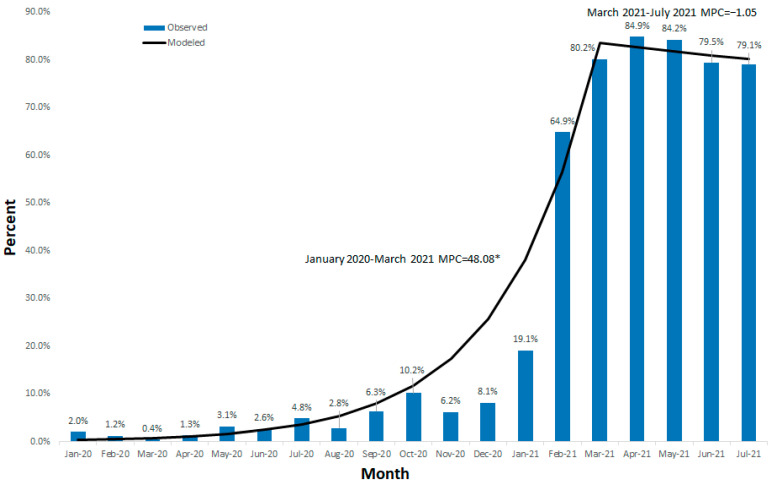
Seropositivity to SARS-CoV-2 between January 2020 and July 2021 in Israel, by month (N = 9520). * *p*-value < 0.05.

Figure 1 demonstrates seropositivity rates between January 2020 and July 2021 by month.

Between January and August 2020, the seropositivity rate was lower than 5.0%, and increased from September (6.3%; 95%CI: 4.4–8.8%) to January 2021 (19.1%; 95%CI: 15.7–22.8%), though fluctuations were observed. In February, the seropositivity rate increased (64.9%; 95%CI: 60.5–69.0%), reached 84.9% (95%CI: 81.4–87.9%) in April, and was stabilized in July (79.1%; 95%CI: 75.4–82.5%). Further analysis performed using Jointpoint to appraise the trends in seropositivity found one breakpoint in March 2021, dividing this study period into two; between January 2020 and March 2021, where a significant increase was observed (MPC = 48.1%; *p*-value < 0.001), and between March and July 2021, where stability was observed (MPC = −1.05%; *p*-value = 0.779) (Figure 1).

Table 2 presents seropositivity rates by demographic characteristics between January and December 2020 (period 1; N = 6042), and January and July 2021 (period 2; N = 3478).

The univariable and multivariable analyses for the association between demographic characteristics and SARS-CoV-2 seropositivity between January and December 2020 are presented in Table 3.

In the multivariable analysis, we have shown that low socio-economic rank was an independent risk factor for seropositivity (OR = 2.49; 95%CI: 1.64–3.80), and residents of the southern district were less likely to be seropositive than residents of the central district (OR = 0.44; 95%CI: 0.22–0.87).

The univariable and multivariable analyses for the association between SARS-CoV-2 seropositivity and demographic characteristics between January and July 2021 are presented in Table 4.

In the multivariable analysis, the 40.00–64.99-year-old age group (OR = 1.36; 95%CI: 1.13–1.64, compared to 16.00–39.99), Jews and others (OR = 1.49; 95%CI: 1.19–1.86, compared to Arabs) and residents of the Northern district (OR = 1.55; 95%CI: 1.09–2.20 compared to Central district) were significantly more likely to be seropositive to SARS-CoV-2.

## 4. Discussion

The aim of the present survey was to characterize and to describe the dynamics of the seropositivity rate to SARS-CoV-2 before and after the introduction of the vaccine in Israel. The results showed that the seropositivity rate to SARS-CoV-2 increased slowly between January and December 2020 due to exposure to the virus; however, following the implementation of the vaccine program in Israel, a significant increase was observed until April 2021, stabilizing thereafter.

We have shown that between January and December 2020, seropositivity rates were between 0.4% (March 2020) and 10.2% (October 2020). During the same year, higher seropositivity rates were observed in New York City (May–July 2020; 18+ years; 23.6% [10] and June–October 2020; 18+ years; 24.3% [11]), Saudi-Arabia (May–July 2020; 18+ years; 19.3%) [12], Ireland (June–July 2020; 18+ years; 12.6%) [13], and Mexico (August–November 2020; 20–39 (27.9%), 40–59 (27.8%) and 60+ (18.6%) years) [14]. Similar rates were observed in Ethiopia (June–July 2020; 15+ years, 3.2%) [15], England (April–September 2020; 18–65 years; 5.9%) [16], Amsterdam, the Netherlands (June and October 2020; 18–70 years, 9.4%) [17], Germany (May–June 2020; 18+ years; 11.3%) [10], and the United States (July 2020 (3.5%), December 2020 (11.5%); 16+ years) [18]. In Australia, between April and June 2020, the seropositivity rate among 20 year olds and above was lower than 1% [19].

The seropositivity rates observed in our study between January and December 2020 corresponded with the extent of the actual exposure of the Israeli population to SARS-CoV-2, and reflects the three COVID-19 waves. The first COVID-19 wave, observed between March and April 2020, was reflected by an increase from 0.4% to 1.2% in seropositivity, respectively; the second, between September and October 2020, was reflected by an increase from 6.3% to 10.2% in seropositivity, respectively; and the third wave started in December 2020 (8.1%) until February 2021 (64.9%). The seropositivity rate is a reliable measure for exposure to SARS-CoV-2 in comparison to the number of confirmed COVID-19 cases reported, affected by the number of PCR tests performed and the testing policy, which changed rapidly throughout the outbreak period. It should be taken into account that the accuracy of the seropositivity obtained using ELISA is high, with 88% sensitivity and 98% specificity, but not perfect [9]. Indeed, we have interestingly shown that in January and February 2020, seropositivity rates were 2.0% and 1.2%, respectively, though officially, the first confirmed case (an imported one) was recorded only by the end of February. A potential explanation may be false positive results as the specificity of the test is 98% [9]; thus, 2% percent false positives are expected.

We have shown that SARS-CoV-2 infection-induced seroprevalence between January and December 2020 was independently associated with low socio-economic rank. Similar findings were also observed in the United States [10,20], in Mexico [14], in a study performed in Colombia among children [21], and in a previous sero-epidemiological survey we conducted among children between January 2020 and March 2021 in Israel [22]. SARS-CoV-2 is transmitted from person to person by exposure to infected respiratory fluids. As the number of contacts increase, the odds for infection become higher, and in a densely populated country such as Israel, high exposure to SARS-CoV-2 should be expected.

According to our data, by December 2020, 8.1% of those aged 16 years and above were exposed to SARS-CoV-2, while based on the national repository of PCR tests, 3.6% were confirmed as infected with SARS-CoV-2 [1]. Using these data, for every confirmed case in Israel, the number of SARS-CoV-2 seropositive cases was 2.2. Similar ratios were observed in the US in July 2020 (3.1) and in May 2021 (2.1) [18], as well as in a previous study we performed among children aged <16 years in Israel, where a ratio of 2.3 was demonstrated in December 2020 [22]. The disparity observed between these rates may be explained by asymptomatic infections, which were not tested and were not confirmed by PCR. Another explanation is the changing PCR test policy in Israel during the outbreak, which may have underestimated the number of those confirmed with SARS-CoV-2.

On December 20, 2020, a wide, aggressive campaign was launched in Israel, aiming to vaccinate the entire 16-year-old and above population against SARS-CoV-2 within a short time period, and by February 2021, 51.3% of the 16-year-old and above population were vaccinated in Israel [4]. We have shown that, in parallel to the introduction of the vaccine in Israel, the seropositivity rate increased significantly by April 2021 and remained constant at 80% until July 2021. Lower seropositivity rates were demonstrated in Los Angeles, California (April 2021; 18+ years; 72.2%) [23], Western Romania (March–June 2021; 18+ years; 45.6%) [24], and in the United States (May 2021; 18+ years; 20.2%) [18]. The seropositivity rates observed after the introduction of the vaccine in Israel reflect the cumulative effect of infection but also of vaccination, which was responsible for the significant increase in seropositivity rates, as the incidence of COVID-19 in those months in Israel was low. The third COVID-19 wave in Israel occurred between December 2020 and March 2021, and the fourth began in June 2021 and continued until October 2021. The parallelization of seropositivity and COVID-19 waves in Israel was impossible, since we could not differentiate between those who were vaccinated and those who were infected, and the rate of those who were infected in these waves was most probably negligible in comparison to the high vaccine acceptance in Israel.

After the introduction of the vaccine, we have shown a significant difference in seropositivity rate by ethnicity as Jews and others were more likely than Arabs to be seropositive. Differences in COVID-19 vaccination according to ethnicity were also demonstrated in the UK [25] and the USA [18,26]. This finding may be explained by the low COVID-19 vaccination rates among the Arab population in Israel, as was previously reported [27,28]. We have also demonstrated that residents of the Northern district were more likely to be seropositive. Geographical differences in seropositivity rates were also observed in the US [20], in Peru [29], and in a previous study we performed among children younger than 16 years [22]. In addition, the 40.00–64.99-year-old age group was more likely than the 16.00–39.99- and 65+-year-old groups to be seropositive. The high odds ratios for seropositivity in these population groups may reflect higher vaccination rates but may also reflect higher exposure to the virus. Since we were not able to differentiate the origin of anti-SARS-CoV-2 antibodies, robust conclusions cannot be drawn.

Future invasions of new variants of concern (VOCs) may cause breakthrough infections and reinfections, leading to difficulty in controlling a new COVID-19 wave. Strategies to mitigate the influence of VOCs in specific populations should be developed accordingly. Using a mathematical model performed to characterize the population-level impact of SARS-CoV-2 variants, it has been shown that the combination of high transmissibility and partial immune/vaccine escape increases not just the total size of the epidemic but also the number of primary infections in susceptible hosts, who are more likely to suffer severe illness or death [30].

The benefits of the study were the systematic, continuous, representative sample collection, the long study period, which reflected three COVID-19 waves in Israel, and the large sample size.

The disadvantages of the study were as follows. 1. The seropositivity rate to SARS-CoV-2 may have been overestimated, since the sample was based on five different laboratories, which perform routine blood tests obtained from patients, mostly with medical backgrounds and may be vulnerable to infection with SARS-CoV-2. 2. We were unable to link the serological result with the national repository of SARS-CoV-2 PCR test results, since the INSB sera samples are anonymous. 3. The inability to differentiate between exposure due to infection or vaccination. 4. Vaccination with SARS-CoV-2 was provided in Israel in an age-descending order with priorities to population groups who were at higher risk, such as the older population and healthcare workers. This gradation was not taken into account in the analysis. 5. Treatment with immunosuppressive medications, such as biologic disease-modifying anti-rheumatic drugs (bDMARDs), can reduce vaccine effectiveness; thus, a lack of drug anamnesis may be considered a major bias.

## 5. Conclusions

Our study results indicate a slight increase in the seropositivity rate to SARS-CoV-2 due to exposure to the virus, but a significant increase following the introduction of the vaccine in Israel. The challenges introduced during the global pandemic and the new emerging VOCs that appeared stress the importance of population-based seroprevalence studies, which provide an estimate of the extent of population protection against future waves, as well as the proportion of those who should be targeted for vaccination. The challenges increase in parallel to the implementation of a “living with COVID-19” strategy, which includes removing most restrictions.

## Figures and Tables

**Table 1:** Demographic characteristics of the total study population and by period (period 1 = January–December 2020 and period 2 = January–July 2021).

		Total		Period 1		Period 2	
	Category	N	%	N	%	N	%
	**Total**	9520	100.0	6042	100.0	3478	100.0
**Age**	**Mean± Standard deviation; Minimum–Maximum**	45.7 ± 21.4; 16.0–103.6		45.8 ± 21.6; 16.0–100.6		45.5 ± 21.0; 16.0–103.6	
**Age group (years)**	**16.00–39.99**	4397	46.2	2796	46.3	1601	46.0
	**40.00–64.99**	2953	31.0	1828	30.3	1125	32.4
	**65.00+**	2167	22.8	1415	23.4	752	21.6
**Gender**	**Male**	4353	45.7	2819	46.7	1534	44.1
	**Female**	5167	54.3	3223	53.3	1944	55.9
**Birth country**	**Israel**	7157	75.3	4471	74.1	2686	77.3
	**Other**	2352	24.7	1563	25.9	789	22.7
**Population group**	**Jews and others**	6215	70.8	3936	70.3	2279	71.8
	**Arabs**	2560	29.2	1665	29.7	895	28.2
**District**	**Jerusalem**	1379	14.5	867	14.4	512	14.8
	**North**	2382	25.1	1461	24.2	921	26.6
	**Haifa**	892	9.4	574	9.5	318	9.2
	**Central**	855	9.0	534	8.9	321	9.3
	**Tel Aviv**	929	9.8	551	9.1	378	10.9
	**South**	2556	26.9	1711	28.4	845	24.4
	**Judea and Samaria**	501	5.3	331	5.5	170	4.9
**Socio-demographic rank**	**High (6–10)**	3090	33.3	1962	33.3	1128	33.2
	**Low (1–5)**	6200	66.7	3925	66.7	2275	66.8

**Table 2:** SARS-CoV-2 seropositivity by demographic characteristics and time periods (period 1 = January–December 2020 and period 2 = January–July 2021).

		Period 1				Period 2			
	Category	Tested	Positive	%	95%CI ^£^	Tested	Positive	%	95%CI ^£^
**Age group (years)**	**16.00–39.99**	2796	116	4.2	3.4–5.0	1601	1093	68.3	65.9–70.6
	**40.00–64.99**	1828	67	3.7	2.8–4.6	1125	835	74.2	71.6–76.8
	**65.00+**	1415	66	4.7	3.6–5.9	752	518	68.9	65.4–72.2
**Gender**	**Male**	2819	115	4.1	3.4–4.9	1534	1095	71.4	69.0–73.6
	**Female**	3223	134	4.2	3.5–4.9	1944	1351	69.5	67.4–71.5
**Birth country**	**Israel**	4471	202	4.5	3.9–5.2	2686	1900	70.7	69.0–72.4
	**Other**	1563	47	3.0	2.2–4.0	789	543	68.8	65.5–72.0
**Population group**	**Jews and others**	3936	152	3.9	3.3–4.5	2279	1643	72.1	70.2–73.9
	**Arabs**	1665	89	5.4	4.3–6.5	895	573	64.0	60.8–67.2
**District**	**Jerusalem**	867	54	6.2	4.7–8.0	512	373	72.8	68.8–76.7
	**North**	1461	66	4.5	3.5–5.7	921	690	74.9	72.0–77.7
	**Haifa**	574	15	2.6	1.5–4.3	318	234	73.6	68.4–78.4
	**Central**	534	14	2.6	1.4–4.4	321	241	75.1	70.0–79.7
	**Tel Aviv**	551	38	6.9	4.9–9.3	378	246	65.1	60.0–69.9
	**South**	1711	43	2.5	1.8–3.4	845	522	61.8	58.4–65.1
	**Judea and Samaria**	331	15	4.5	2.6–7.4	170	130	76.5	69.4–82.6
**Socio-demographic rank**	**High (6–10)**	1962	40	2.0	1.5–2.8	1128	853	75.6	73.0–78.1
	**Low (1–5)**	3925	203	5.2	4.5–5.9	2275	1569	69.0	67.0–70.9

^£^ CI—Confidence interval.

**Table 3:** Univariable and multivariable analyses for the association between SARS-CoV-2 seropositivity and demographic characteristics in Israel, period 1 (January–December 2020).

		Univariable			Multivariable		
	Category	OR ^€^	95%CI ^£^	*p*-Value	OR ^€^	95%CI ^£^	*p*-Value
**Age** **group (years)**	**16.00–39.99**	Ref.					
	**40.00–64.99**	0.88	0.65–1.20	0.4099			
	**65.00+**	1.13	0.83–1.54	0.4373			
**Gender**	**Male**	0.98	0.76–1.26	0.8789			
	**Female**	Ref.					
**Birth country**	**Israel**	1.53	1.10–2.11	0.0102	1.25	0.87–1.80	0.2242
	**Other**	Ref.			Ref.		
**Population group**	**Jews and others**	Ref.			Ref.		
	**Arabs**	1.41	1.08–1.84	0.0127	1.30	0.89–1.91	0.1771
**District**	**Jerusalem**	2.47	1.36–4.49	0.0031	1.06	0.54–2.08	0.8764
	**North**	1.76	0.98–3.16	0.0591	0.76	0.39–1.48	0.4154
	**Haifa**	1.00	0.48–2.08	0.9929	0.70	0.32–1.50	0.3566
	**Central**	Ref.			Ref.		
	**Tel Aviv**	2.75	1.47–5.14	0.0015	1.71	0.87–3.35	0.1175
	**South**	0.96	0.52–1.76	0.8893	0.44	0.22–0.87	0.0190
	**Judea and Samaria**	1.76	0.84–3.70	0.1340	1.05	0.48–2.29	0.9076
**Socio-demographic rank**	**High (6–10)**	Ref.			Ref.		
	**Low (1–5)**	2.62	1.86–3.69	<0.0001	2.49	1.64–3.80	<0.0001

^€^ OR—Odds Ratio; ^£^ CI—Confidence interval.

**Table 4:** Univariable and multivariable analyses for the association between SARS-CoV-2 seropositivity and demographic characteristics in Israel, period 2 (January–July 2021).

		Univariable			Multivariable		
	Category	OR ^€^	95%CI ^£^	*p*-Value	OR ^€^	95%CI ^£^	*p*-Value
**Age group (years)**	**16.00–39.99**	Ref.					
	**40.00–64.99**	1.34	1.13–1.59	0.0008	1.36	1.13–1.64	0.0013
	**65.00+**	1.03	0.85–1.24	0.7653	1.02	0.82–1.25	0.8899
**Gender**	**Male**	1.10	0.94–1.27	0.2267			
	**Female**	Ref.					
**Birth country**	**Israel**	1.10	0.92–1.30	0.3005			
	**Other**	Ref.					
**Population group**	**Jews and others**	1.45	1.23–1.71	<0.0001	1.49	1.19–1.86	0.0005
	**Arabs**	Ref.					
**District**	**Jerusalem**	0.89	0.65–1.23	0.4777	1.15	0.79–1.68	0.4701
	**North**	0.99	0.74–1.33	0.9548	1.55	1.09–2.20	0.0140
	**Haifa**	0.92	0.65–1.32	0.6659	1.08	0.74–1.59	0.6890
	**Central**	Ref.					
	**Tel Aviv**	0.62	0.44–0.86	0.0043	0.71	0.49–1.02	0.0601
	**South**	0.54	0.40–0.72	<0.0001	0.85	0.60–1.20	0.3480
	**Judea and Samaria**	1.08	0.70–1.67	0.7329	1.32	0.82–2.11	0.2525
**Socio-demographic rank**	**High (6–10)**	1.40	1.19–1.64	<0.0001	1.23	0.99–1.53	0.0607
	**Low (1–5)**	Ref.					

^€^ OR—Odds Ratio; ^£^ CI—Confidence interval.

## Data Availability

Not applicable.
